# T Cells That Help B Cells in Chronically Inflamed Tissues

**DOI:** 10.3389/fimmu.2018.01924

**Published:** 2018-08-23

**Authors:** Deepak A. Rao

**Affiliations:** Division of Rheumatology, Immunology, Allergy, Department of Medicine, Brigham and Women's Hospital, Harvard Medical School, Boston, MA, United States

**Keywords:** T follicular helper cells, T peripheral helper cells, IL-21, CXCL13, B cells, ectopic lymphoid structure, ectopic lymphoid follicle, tertiary lymphoid tissue

## Abstract

Chronically inflamed tissues commonly accrue lymphocyte aggregates that facilitate local T cell-B cell interactions. These aggregates can range from small, loosely arranged lymphocyte clusters to large, organized ectopic lymphoid structures. In some cases, ectopic lymphoid structures develop germinal centers that house prototypical T follicular helper (Tfh) cells with high expression of Bcl6, CXCR5, PD-1, and ICOS. However, in many chronically inflamed tissues, the T cells that interact with B cells show substantial differences from Tfh cells in their surface phenotypes, migratory capacity, and transcriptional regulation. This review discusses observations from multiple diseases and models in which tissue-infiltrating T cells produce factors associated with B cell help, including IL-21 and the B cell chemoattractant CXCL13, yet vary dramatically in their resemblance to Tfh cells. Particular attention is given to the PD-1^hi^ CXCR5^−^ Bcl6^low^ T peripheral helper (Tph) cell population in rheumatoid arthritis, which infiltrates inflamed synovium through expression of chemokine receptors such as CCR2 and augments synovial B cell responses via CXCL13 and IL-21. The factors that regulate CD4^+^ T cell production of CXCL13 and IL-21 in these settings are also discussed. Understanding the range of T cell populations that can provide help to B cells within chronically inflamed tissues is essential to recognize these cells in diverse inflammatory conditions and to optimize either broad or selective therapeutic targeting of B cell-helper T cells.

## Introduction

CD4^+^ T cells play a critical role in stimulating effective B cell responses and production of high-affinity antibodies. T follicular helper (Tfh) cells are generally considered the dominant T cell population capable of providing help to B cells. The interactions between Tfh cells and B cells within follicles of secondary lymphoid organs (SLOs) occur with precise spatial and temporal coordination to yield productive antibody responses. Yet in both protective and pathologic immune responses, T cell-B cell interactions also occur outside of SLOs and within inflamed peripheral tissues. Interactions between T cells and B cells within peripheral tissues are much less well characterized, and the T cell populations that participate in these interactions can differ from Tfh cells in their surface features, migration patterns, and effector functions. This review examines the phenotypes of T cells within peripheral tissues that express factors associated with providing B cell help, in particular IL-21 and CXCL13. In many cases, the T cells that help B cells within peripheral tissues do not conform to the typical phenotype of active Tfh cells and often lack the signature features of Tfh cells such as high expression of CXCR5 and Bcl6. These observations underscore that a broader definition of B cell-helper T cells is required to fully capture the range of T cells capable of productive T cell-B cell interactions, in particular during pathologic chronic immune responses.

## The T follicular helper paradigm

The discovery and characterization of Tfh cells has helped define the paradigm of productive T cell-B cell interactions and has established Tfh cells as the prototype of a B cell-helper T cell [1]. Tfh cells are drawn into lymphoid follicles through expression of CXCR5, a chemokine receptor shared with B cells that detects the chemokine CXCL13 [2–4]. Expression of CXCR5 is a defining feature of Tfh cells, a logical association given that this chemokine receptor helps direct these cells to the location after which they are named [5–8]. The transcription factor Ascl2 enables early CXCR5 expression, which is then reinforced by the central Tfh transcription factor Bcl6 [9–13]. Upregulation of CXCR5 and downregulation of CCR7 induces migration of CXCR5^+^ T cells into follicles, where interactions with B cells enhance Bcl6 expression and stabilize the Tfh cell phenotype, in part through ICOS-ICOSL interactions [10, 14–16].

Bcl6 is essential for the development and persistence of Tfh cells *in vivo* and promotes expression of many Tfh cell-associated factors, including CXCR5, ICOS, PD-1, and CXCL13, while suppressing alternative differentiation paths [10–13, 17]. Human but not mouse Tfh cells produce large amounts of CXCL13, which helps to recruit CXCR5^+^ B cells to follicles [13, 18, 19]. In addition, Tfh cells characteristically express IL-21, a cytokine that promotes B cell proliferation in germinal centers (GC) and differentiation into plasma cells [20–22]. While there is heterogeneity in Tfh cell phenotypes and functions in SLOs, GC-Tfh display the most pronounced B cell-helper phenotype, with high expression of CXCR5, Bcl6, CXCL13, and IL-21 accompanied by high expression of the immunomodulatory receptors ICOS and PD-1 [1, 18, 20, 23]. These 4 key features of GC-Tfh cells: (1) CXCR5 expression, (2) high Bcl6 expression, (3) surface expression of PD-1 and ICOS, and (4) secretion of IL-21 and CXCL13, are commonly assayed in studies looking for Tfh-like cells at sites outside of SLOs, including blood and peripheral tissues.

## T cell-B cell interactions in inflamed tissues

During an adaptive immune response, activated T cells differentiate into distinct effector populations that acquire specialized functions coupled with appropriate migratory programs. For example, activated effector or effector memory cells home to peripheral tissues to direct inflammatory responses, while CXCR5^+^ Tfh cells migrate to lymphoid follicles to help B cells [24]. Migratory capacity sometimes serves as a defining feature of T cell populations: CCR7^+^ CD62L^+^ T central memory cells recirculate through SLOs, CCR7^−^ CD62L^−^ T effector memory cells traffic through peripheral tissues, and CD103^+^ CD69^+^ T resident memory cells localize to tissue barriers [25]. However, in pathologic conditions involving chronic inflammation, such as autoimmune diseases, cancer, and organ transplantation, the anatomic distinction between inflamed peripheral tissues and lymph node follicles begins to blur. Chronically inflamed sites frequently develop aggregates of T cells and B cells that promote B cell responses locally within the tissue [26]. Often these aggregates appear as small, disorganized lymphocyte clusters. In some cases, the aggregates mature into organized ectopic lymphoid structures (ELS, also referred to as tertiary lymphoid organs/tissues/structures) that acquire many features of follicles in SLOs, including compartmentalization of T cell-rich and B cell-rich zones and accumulation of follicular dendritic cells (FDC) [26].

T cell-B cell interactions within chronically inflamed tissues can reproduce many of the key features of productive interactions within SLO follicles, including somatic hypermutation, class switching, and differentiation of plasma cells [26]. For example, the inflamed synovium in rheumatoid arthritis (RA) develops lymphoid aggregates, which can range from small clusters to organized follicles with GCs [27]. Plasma cells differentiate within these aggregates and are often seen extending out from the borders of the aggregates [28, 29]. Similarly, somatic hypermutation and differentiation of plasmablasts occurs within tubulointerstitial aggregates in kidneys affected by lupus nephritis [30]. Infiltrated tumors and rejecting kidney allografts also show evidence of lymphoid aggregates that support B cell somatic hypermutation despite the absence of typical GC [31–34]. The accumulation of lymphocytes and plasma cells in chronically inflamed tissues occurs frequently enough to have merited its own term “lymphoplasmacytic infiltrate,” which appears not uncommonly in clinical histopathologic reports.

Defining the T cell populations most relevant for driving B cell aggregation and proliferation within peripheral tissues remains challenging. It has been generally assumed that Tfh cells infiltrate peripheral tissues to drive B cell responses within these tissues. However, this assumption requires some caution. For one, the migratory receptors required to infiltrate a peripheral tissue differ substantially from those required to access SLOs. CXCR5^+^ Tfh cells typically do not express chemokine receptors that recruit T cells to inflamed peripheral tissues, such as CCR2, CCR5, and CX3CR1 [1]. Rather, a tightly controlled migratory program helps restrict Tfh cells to CXCL13-laden follicles. Thus it is not obvious how Tfh cells would be initially recruited to inflamed sites that lack well-established follicles. This raises the possibility that T cells with a distinct migratory capacity—directed by expression of a distinct cohort of migratory receptors—may interact with B cells in diffusely inflamed tissues.

Second, lymphocyte populations within inflamed tissues are dominated by memory cells that have undergone prior activation, with a smaller representation of naïve lymphocytes than in SLOs. Both memory T cells and B cells differ in their responsiveness to antigen-receptor activation, requirement for costimulation, and sensitivity to cytokines and other inputs as compared to their naïve counterparts [25, 35, 36]; thus, the interactions between effector/memory T cells and B cells within inflamed tissues may differ from those that occur within follicles of SLOs. Even within the same tonsil, different B cell populations (naïve, memory, GC-B cells) yield markedly different responses when co-cultured with distinct tonsil CD4^+^ T cell populations: GC-Tfh strongly promote immunoglobulin production from GC-B cells, yet ICOS^low^ CXCR5^low^ T cells fail to do so because the FasL that they produce kills co-cultured GC-B cells [23]. In contrast, naïve and memory B cell populations respond well to CXCR5^low^ ICOS^low^ T cells because these B cell populations do not express Fas and are insensitive to FasL [23]. Thus the rules of engagement may differ depending on the specific cell types present and the organization of the interactions.

Finally, determining the phenotype of T cells that help B cells in inflamed tissues faces technical challenges. The tissues that can be obtained are often small, with many fewer lymphocytes available compared to SLOs. In addition, the highly overlapping expression programs between Tfh cells and B cells limit interpretation of total tissue sample analyses (e.g., mRNA from the whole tissue) because one cannot distinguish whether key factors such as CXCR5 and Bcl6 are derived from the T cells or B cells. The tightly entangled interaction between T cells and B cells in tissues provides challenges for standard immunofluorescence microscopy and even laser capture microscopy [37]. Immunohistochemistry and immunofluorescence microscopy allow visualization of T cell-B cell aggregates and may provide the resolution to discriminate T cell vs. B cell phenotypes. However, relatively few parameters are measured simultaneously, and discriminating cell surface protein expression (e.g., CXCR5 expression) on the surface of T cells surrounded by a tight cluster of CXCR5-bright B cells can be difficult. In addition, the cellular source of soluble factors such as IL-21 and CXCL13 can be ambiguous, especially for factors that bind to extracellular matrix [38–40]. Utilization of single cell resolution analyses are therefore particularly valuable to interrogate in detail the phenotypes of potential T cells that help B cells within tissue samples.

## B cell-helper T cells IN rheumatoid arthritis

RA synovium provides a valuable case study to interrogate T cell-B cell interactions in a chronically inflamed tissue. RA is an autoimmune disease that prominently features pathologic T cell-B cell interactions. Approximately 2/3 of RA patients develop autoantibodies—antibodies against citrullinated proteins and/or other immunoglobulins (rheumatoid factors). Seropositive RA patients commonly develop T cell-B cell aggregates within synovial tissue [41]. These are often small or medium-sized aggregates, although ~10–15% of patients develop more organized aggregates with features of GC [27, 42, 43]. Most B cells in RA synovium are CD27+ memory B cells with mutated B cell receptor sequences, although a minority of naïve B cells can also be found within lymphoid aggregates [28, 44, 45]. Synovial B cells produce RA-associated autoantibodies, and B cell receptor repertoire analyses suggest that memory B cells activated in synovial aggregates can differentiate into plasma cells locally within the tissue, even in the absence of GCs [28, 29, 46]. Plasma cells are often found extending out from around lymphoid aggregates [29]. In some patients, plasma cells accumulate densely throughout the synovium, which is a defining feature of a highly inflamed RA synovium and can distinguish RA from other causes of early arthritis [47, 48].

Given the prominent lymphocyte aggregates and evidence of ongoing B cell activation, RA synovium appears a very likely place for accumulation of Tfh cells to help drive these responses. RA is somewhat unique among human autoimmune diseases in that relatively large samples of the target tissue can be obtained for research. Synovial tissue obtained either at the time of joint replacement surgery or through a research-protocol biopsy can provide sufficient material for multiple high-dimensional cellular analyses [41, 49]. Surprisingly, a mass cytometry screen of T cells isolated from RA synovial tissue performed by our group revealed few CXCR5^+^ Tfh cells in RA synovium, despite frequent B cells and plasma cells [50]. In addition, flow cytometry demonstrated an almost total absence of Tfh cells in RA synovial fluid. However, seropositive RA synovial tissue and fluid samples contained a large population of PD-1^hi^ CXCR5^−^ T cells, comprising ~25% of the CD4^+^ T cells, that expressed high levels of IL-21 and CXCL13 and induced memory B cell differentiation into plasma cells *in vitro*. CXCR5 expression was absent from PD-1^hi^ CXCR5^−^ cells at both the protein and mRNA level, in contrast to the well-detected CXCR5 expression in tonsil Tfh cells and circulating Tfh cells. PD-1^hi^ CXCR5^−^ T cells were observed adjacent to B cells both within lymphocyte aggregates and more diffusely throughout inflamed synovium. Like Tfh cells, synovial PD-1^hi^ CXCR5^−^ T cells also expressed high levels of ICOS and MAF, a transcription factor that promotes IL-21 production (discussed below). Cytometric and transcriptomic comparisons of PD-1^hi^ CXCR5^−^ and PD-1^hi^ CXCR5^+^ cells from blood showed high expression of TIGIT, SAP, CD200, and SLAM and low expression of CD25 and CD127 in both populations [50].

However, PD-1^hi^ CXCR5^−^ T cells also showed key differences from Tfh cells. Unlike Tfh cells, PD-1^hi^ CXCR5^−^ cells from synovium did not express high levels of Bcl6, and instead they showed elevated levels of the counter-regulator Blimp1, which opposes the actions of Bcl6 [10]. In addition, PD-1^hi^ cells from synovium not only lacked CXCR5, but they frequently expressed a different cohort of chemokine receptors, including CCR2, CCR5, and CX3CR1, with approximately half of the PD-1^hi^ cells expressing CCR2 [50]. Notably, these receptors are abundant on leukocytes that infiltrate RA synovium, which contains high levels of the CCR2 ligand CCL2 (MCP-1) and the CCR5 ligand RANTES, among others [51–55]. These chemokines are so prominent in inflamed synovium that attempts have been made to interfere with both CCR2- and CCR5-mediated trafficking therapeutically to blunt synovium inflammation, although such approaches have not yet succeeded perhaps due to migratory signal redundancy or incomplete signal blockade [56–58].

## Peripheral vs. follicular B cell-helper T cells

**Figure 1:**
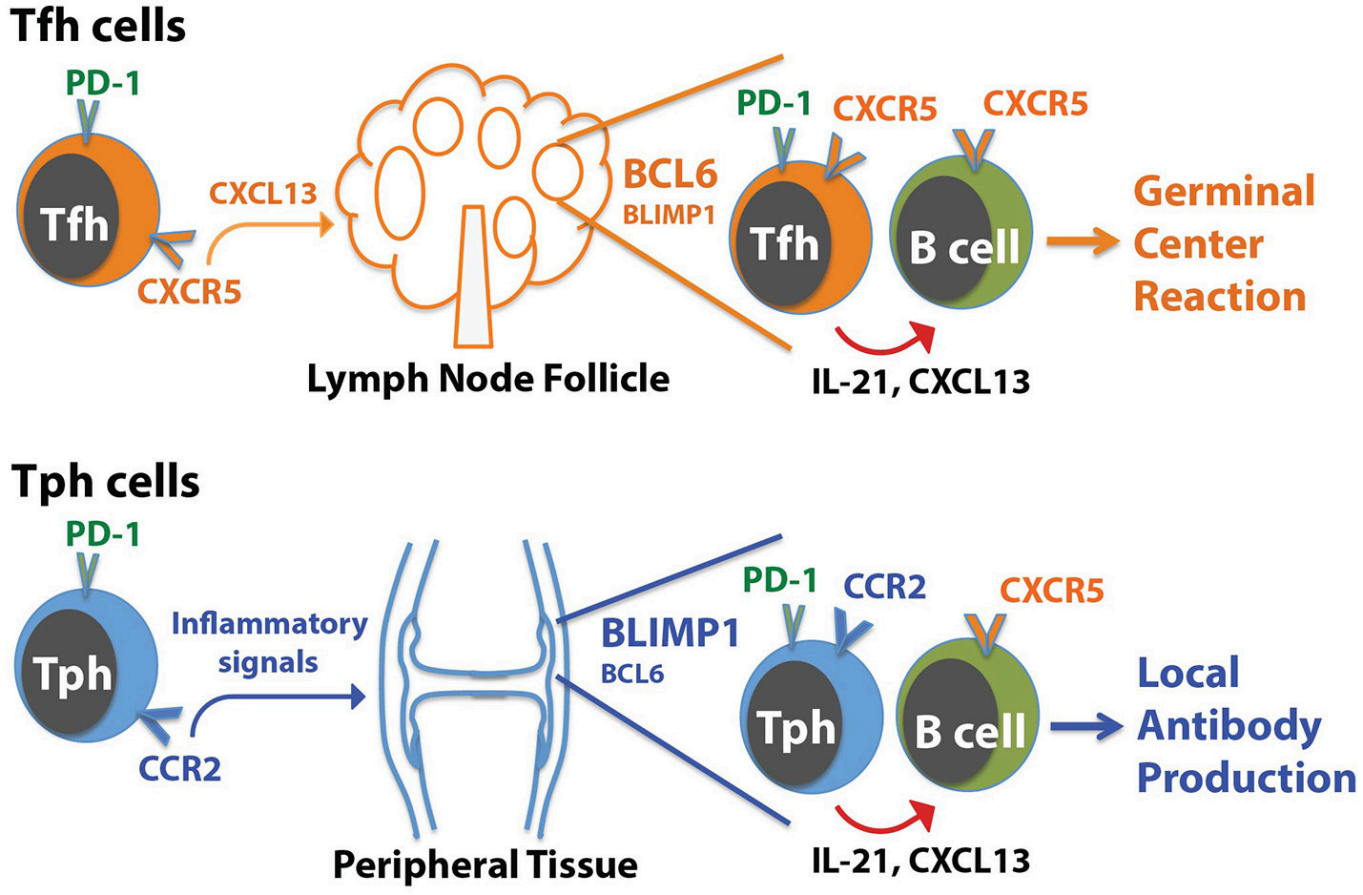
Distinctions between Tph cells and Tfh cells. Tfh cells home to follicles following the chemokine CXCL13, which acts on CXCR5 on the Tfh cells. In contrast, Tph cells express a distinct set of chemokine receptors that direct migration to sites of inflammation, such as the RA joint. In both cases, Tph cells or Tfh cells interact with CXCR5+ B cells by producing CXCL13 to help recruit B cells, and IL-21 to help drive B cell activation and differentiation. For Tfh cells, this promotes generation of a GC reaction in follicles. For Tph cells, this may augment local antibody production within the tissue.

The presence of a B cell-helper T cell population in RA synovium with a CXCR5^−^ Bcl6^low^ but CCR2^+^ Blimp1^+^ phenotype illustrates that IL-21, CXCL13, and B cell help can be provided by T cell populations that differ substantially in phenotype from Tfh cells (Figure 1). Two key points arise from these observations:

1. *T cells that help B cells do not necessarily express CXCR5*. The RA synovium observations reveal a population of T cells that home to sites of inflammation, using chemokine receptors that target these sites, and help B cells within these sites. We have suggested that these CXCR5^−^ PD-1^hi^ T cells can be considered peripheral, rather than follicular, B cell-helper T cells, and we referred to these cells as T peripheral helper (Tph) cells for shorthand [50]. Once activated within inflamed tissues, Tph cells may secrete CXCL13 to recruit B cells (and potentially Tfh cells) and produce IL-21 to help drive B cell survival and maturation.
2. *T cells that help B cells do not necessarily express high levels of Bcl6*. The Tph cells in RA synovium express modest levels of Bcl6, well below that seen in synovial CXCR5^+^ PD-1^hi^ Tfh cells, indicating that B cell-helper function can be dissociated from high Bcl6 expression. As discussed below, Bcl6 does not directly control production of IL-21 [11, 13]. Rather, one major role for high Bcl6 levels is directing the positioning of Tfh cells in follicles through upregulation of CXCR5 and downregulation of CCR7, PSGL1, and EBI2 [13, 17]. High Bcl6 expression also helps CXCR5+ cytotoxic CD8+ T cells localize to follicles [59]. This role of Bcl6 in directing T cells to follicles may be less important for B cell-helper T cells that are present in more diffusely infiltrated tissues without true GC. Bcl6 also importantly controls additional features of Tfh cells, including expression of molecules involved in interacting with B cells (ICOS, PD-1, CD40L); however, it is possible that the lower levels of Bcl6, such as seen in synovial Tph cells, may be sufficient for this function. Other transcriptions factors such as MAF may also co-regulate some of these molecules [13].

B cell-helper T cells have been reported within target tissues in range of autoimmune, inflammatory, and malignant conditions; it will be of substantial interest to now clarify the extent to which these cells resemble Tfh cells vs. Tph cells in each condition. There is no doubt that Tfh cells play a critical role in providing help to B cells in SLOs, and it is clear Tfh cells infiltrate well-formed ELS with GC in inflamed tissues as well. Yet the following sections highlight selected diseases and models in which evidence suggests the presence of potential B cell-helper T cells—identified by production of IL-21 or CXCL13—that differ from prototypical Tfh cells. For this discussion, Tfh cells are defined as CXCR5^+^ Bcl6^+^ CD4^+^ T cell that produce IL-21 (and CXC13 in humans), while cells that lack CXCR5 or Bcl6 are considered non-Tfh cells and may represent candidate Tph cells.

## IL-21-producing T cells in inflamed tissues

Tfh cells produce high levels of IL-21 to stimulate B cell survival and differentiation, thus production of IL-21 is often used as a surrogate marker for Tfh cells. It is important to note that IL-21 can also be produced by other CD4^+^ T cell populations and NKT cells, and that this cytokine acts on many different target cell populations to enhance or tailor responses, for example boosting cytotoxicity of CD8^+^ T cells and NK cells, or altering macrophage function [22]. Nonetheless, it seems likely that T cells that produce IL-21 while in close proximity to B cells, for example within lymphocyte aggregates, can enhance B cell responses. The following examples suggest that IL-21-producing CD4^+^ T cells in inflamed tissues can show substantial variability in their expression of Tfh-associated factors.

Transplanted renal and cardiac allografts undergoing rejection can develop ELS with plasma cells that produce donor-specific antibodies [60, 61]. Renal allografts that undergo rapid rejection contain high mRNA expression of IL-21 [62]. IL-21 expression levels correlate strongly with expression of AID, a protein required for somatic hypermutation and class switch recombination in B cells, suggesting a potential connection between T cell-derived IL-21 and B cell activation with the grafts [62]. However, IL-21 expression levels in rejected allografts showed no correlation with Bcl6 expression levels, and fewer than 5% of the CD4^+^ T cells in rejected allografts expressed CXCR5^+^ by flow cytometry [62]. An independent series of analyses of kidney allografts undergoing acute T cell-mediated rejection found that the majority of CD4^+^ T cells within lymphocyte aggregates expressed IL-21 by immunofluorescence microscopy, yet few of these T cells co-expressed Bcl6 [63, 64]. These observations suggest that CD4^+^ T cells within lymphocyte aggregates of rejecting allografts produce abundant IL-21, yet the majority of this IL-21 appears to come from non-CXCR5^+^/Bcl6^+^ CD4^+^ T cells.

In multiple sclerosis, an autoimmune disease that involves infiltration of T cells and B cells in parenchymal demyelinating lesions, as well as development of lymphoid aggregates in the meninges, a similarly high frequency of IL-21-producing T cells has been reported [65–67]. Immunofluorescence microscopy analyses suggested that more than half of CD4^+^ T cells in both active and chronic parenchymal lesions produce IL-21, while the receptor for IL-21 is expressed broadly on infiltrating B cells and T cells and on neurons [67]. The frequency of CXCR5^+^ T cells in these lesions is not yet clear; however, in cerebrospinal fluid from multiple sclerosis patients ~20% of memory CD4^+^ T cells express CXCR5, comparable to the frequency observed in blood. While the expression of CXCR5 may differ between cerebrospinal fluid and tissue, these observations raise the possibility that a large portion of the IL-21^+^ CD4^+^ T cells in multiple sclerosis may not express CXCR5.

Analyses of nasal polyps that develop due to chronic upper airway inflammation indicated that ~20–40% of CD4^+^ T cells produce IL-21, as observed by both flow cytometry and immunohistochemistry [68–70]. CXCR5^−^ effector memory T cells comprise the majority of the IL-21-producing cells in affected nasal tissues [69]. These cells frequently express PD-1 and co-produce IFN-γ [69, 70]. CXCR5^+^ Bcl6^+^ Tfh cells are enriched in the lymphoid aggregates that form in these polyps compared to healthy tissue; however, Bcl6 expression is relatively infrequent in nasal polyps overall (perhaps 2–5% of CD4^+^ T cells), with higher frequencies in T cells from polyps that contained GCs or eosinophils [68–70]. Thus while it is clear that Tfh cells are enriched in nasal polyps, it is not yet clear that Tfh cells provide the majority of the B cell help in nasal polyps.

In contrast to RA and the conditions above, IgG4-related disease (IgG4-RD) provides an example in which *bona fide* GC-Tfh cells appear to dominate the T cell infiltrate. IgG4-RD involves formation of large, fibrotic lesions that are heavily infiltrated by IgG4^+^ plasma cells [71]. The lesions can form in several tissues including the salivary glands (sialadenitis) and lacrimal glands (dacryoadenitis) and contain large ELS with GC [72]. The majority of T cells purified from these lesions show a prototypical GC-Tfh phenotype, with expression of CXCR5, Bcl6, PD-1, ICOS, CXCL13, and IL-21 at high levels that equal or exceed that seen in human tonsils [72, 73]. Interestingly, GCs appear more common in IgG4-RD than in Sjogren's syndrome, an autoimmune disease that also produces lymphoid aggregates in the salivary glands but involves at least in part a CCR9^+^ B cell-helper T cell population with features distinct from Tfh cells (discussed below) [72, 74].

Murine models also support the idea that non-Tfh cells can provide important help to B cells via IL-21. In lupus-prone MRL/lpr mice, a population of CXCR4^+^ PSGL-1^low^ CD4^+^ T cells accumulates within extrafollicular foci in the spleen to support plasmablast responses [75]. These extrafollicular helpers require Bcl6 and ICOS yet express little CXCR5 and do not transmigrate to CXCL13; rather, they express CXCR4, which may help position them in areas of higher CXCL12 concentration such as near the red pulp [75–77]. These extrafollicular helper T cells provide a unique source of IL-21 to promote plasma cell generation and IgG production from B cells in extracellular follicles [75–77]. A potentially comparable T cell population has also been observed in human tonsil, identified as CXCR5^low^ ICOS^low^ CD4^+^ T cells, although it is difficult to fully distinguish whether these cells are extrafollicular helpers or ‘pre-Tfh’ cells [23]. *In vitro*, this CXCR5^low^ ICOS^low^ Bcl6^low^ population efficiently helps naïve and memory B cells via production of IL-21, yet appears to kill, rather than help, GC-B cells via Fas-FasL interactions [23].

Productive interactions between B cells and non-Tfh cells also occur in inflamed peripheral tissues in murine models. In a chronic lung inflammation model induced by ovalbumin + LPS, large lymphoid aggregates develop in the lungs [78]. While occasional aggregates show features of ELS, most appear loose and poorly organized. Interestingly, B cells with GC features, including staining with peanut agglutinin and the monoclonal antibody GL7, were found both in the ELS and in the loose aggregates, suggesting development of GC-like B cells within the lung aggregates; however, no Bcl6^+^ or CXCR5^+^ T cells were present in these tissues [78]. Rather, high IL-21 production and B cell-helper activity existed within the CXCR5^−^ Bcl6^−^ T cell population resident in the lung. While no defined markers were identified to specifically isolate the B cell-helper T cells out of the total lung T cell pool, high average expression of PD-1 and ICOS suggest a likely overlap with the Tph cell described in RA synovium. A separate study of a house dust mite-induced murine asthma model also identified a PD-1^hi^ CXCR5^−^ IL-21-producing CD4^+^ T cell population that accumulates in the lungs [79]. This T cell population derived from Tfh cells in lymph nodes and migrated to the lungs to enhance eosinophilic airway inflammation via IL-21.

Observations from the non-obese diabetic (NOD) mouse provide further evidence of B cell help from non-Tfh cells. NOD mice develop lymphoid aggregates in the pancreas, with development of more organized ELS with FDC networks over time [80]. The pancreas in NOD mice is infiltrated with an IL-21-producing effector memory CD4^+^ T cell population with high ICOS^+^ expression. Strikingly, the majority of these cells expressed CCR9, a chemokine receptor that confers sensitivity to the chemokine CCL25 and helps mediate migration to mucosal sites such as the small bowel [81, 82]. Pancreatic CCR9^+^ T cells expressed high levels of Bcl6 and MAF, yet little CXCR5 and SAP and variable PD-1. Consistent with IL-21 production, pancreatic CCR9^+^ T cells could enhance immunoglobulin production from co-cultured B cells *in vitro* [82]. IL-21^+^ CCR9^+^ cells were also detected in the salivary glands of NOD mice [82]. Consistent with these observations, CCR9^+^ ICOS^+^ cells are present in salivary glands of patients with Sjogren's syndrome, and the levels of the CCR9 ligand CCL25 in salivary tissue correlated positively with the levels of IL-21 and plasma cells in the tissue [83]. In addition, human CCR9^+^ T cells from blood produced IL-21 at even higher levels than do CXCR5^+^ T cells and enhanced responses of co-cultured B cells *in vitro* [81, 83].

An independent study of NOD mice identified a potentially distinct population of B cell-helper T cells that is highly enriched in infiltrated salivary glands. This T cell population, identified as PD-1^hi^ ICOS^hi^ CD73^hi^ CD200^hi^, produced high levels of IL-21 and IFN-γ, suggesting possible B cell-helper function, yet lacked Bcl6 and CXCR5 and also showed low expression of CCR9 [84]. Viewed together, these observations suggest the tissues of Sjogren's syndrome patients and NOD mice accumulate IL-21-producing B cell-helper T cell populations that can lack typical Tfh-associated features such as Bcl6 and CXCR5.

Direct interrogation of Bcl6-deficient T cells provides further support for effective B cell help from non-Tfh cells. In a murine influenza model, Th1 CD4^+^ T cells can promote generation of neutralizing antibodies when Tfh cells are rendered absent due to CD3-specific deletion of Bcl6 [85]. In this model, CXCR5^−^ Th1 cells secreted enough IL-21, co-produced with IFN-γ, to stimulate production of protective, albeit low-affinity, neutralizing antibodies to influenza virus. Bcl6-independent production of IL-21 by CD4^+^ T cells has also been observed in a murine *Plasmodium chabaudi* infection model, in which CD4-specific deletion of Bcl6 eliminated development of CXCR5^hi^ GC-Tfh cells but did not alter production of IL-21 in the spleen, which derived primarily from IL-21/IFN-γ double producers that retained intermediate CXCR5 expression [86].

## Regulation of IL-21 production in T cells

The above examples demonstrate that populations of T cells with varying degrees of similarity to Tfh cells can produce IL-21 and provide B cell help in inflamed tissues. The factors that regulate the ability of CD4^+^ T cells to produce IL-21 differ somewhat between human T cells and mouse T cells. In mice, IL-6 and IL-21 potently induce IL-21 production from T cells stimulated *in vitro*, and IL-21 is frequently detected in cultured of T cells stimulated under Th17-polarizing conditions [22, 87–89]. In contrast, IL-6 and IL-21 do not efficiently induce IL-21 production by human CD4^+^ T cells; IL-12 is far more potent in promoting IL-21 production by human T cells and often induces IFN-γ/IL-21 co-producers [90, 91]. IL-12 can also enhance IL-21 production by murine CD4^+^ T cells, which transit through an early Tfh-like phenotype when stimulated under Th1-inducing conditions [92]. In addition, IL-12 transiently enhances expression of Bcl6, CXCR5, and ICOS via STAT4; this effect is subsequently suppressed by Tbet [92]. The overlap between Tfh and Th1 features in T cells early after activation appears to fit well with the theme that IL-21-producing T cells in inflamed tissues often also show Th1 features and co-produce IFN-γ.

Importantly, transcriptional control of IL-21 production occurs largely independently of Bcl6. Murine CD4^+^ T cells that lack Bcl6 produce IL-21 at normal levels, and overexpression of Bcl6 in murine T cells does not increase IL-21 production [11]. Similarly, overexpression of Bcl6 in human tonsil CD4^+^ T cells does not alter IL-21 production [13]. Instead, the transcription factor MAF, which is highly expressed in both Tfh cells and synovial Tph cells, appears most directly associated with IL-21 production. *In vitro*, MAF can activate an IL-21 promoter-luciferase reporter construct, unlike Tbet or GATA3, and overexpression of MAF in human tonsil CD4^+^ T cells or mouse naïve CD4^+^ T cells increases IL-21 production [13, 93, 94]. In addition, genetic deletion of MAF reduces IL-21 production from murine Th17 cells [95]. Interestingly, in human T cells, the combination of IL-12 plus TGF-β induces several features of Tfh cells, including increased expression of IL-21, MAF, Bcl6, and CXCR5 [91]. This occurs as TGF-β directs the actions of STAT4 and STAT3, activated by IL-12, to enhance Tfh cell features [91]. TGF-β also induces MAF in murine CD4^+^ T cells, yet it concurrently inhibits IL-21 production and does not promote a Tfh phenotype in murine T cells [91, 93]. A more comprehensive delineation of the signals that control IL-21 production, along with other factors required for cell-surface interactions with B cells, will be valuable to better understand the regulation of T cell-B cell interactions in the periphery.

## CXCL13-producing T cells in inflamed tissues

While IL-21 can be produced broadly by different T cell populations and may act on diverse target cells, production of CXCL13 appears to be a much more specific marker of T cell-B cell interactions. CXCL13 acts specifically to recruit and position CXCR5^+^ cells within lymphoid follicles, primarily B cells and Tfh cells [2–4, 8, 96]. Overexpression of CXCL13 in a peripheral tissue induces formation of B cell aggregates that eventually mature into ELS, while neutralization of CXCL13 in murine models reduces lymphoid aggregate formation and tissue inflammation severity in several chronic inflammation models [97–103]. Consistent with these observations, the highest expression of CXCR5 in RA synovium is found on B cells, with bright CXCR5 expression seen on B cells within synovial aggregates [50].

Importantly, Tfh cells from humans and primates, but not rodents, produce large amounts of CXCL13 [1, 7, 18]. In lymph nodes, GC-Tfh cells are an important, and perhaps primary, source of CXCL13. Immunohistochemistry of tonsils shows co-staining of CXCL13 primarily with PD-1^+^ T cells, rather than CD21^+^ FDCs, within follicles [104, 105]. Intracellular flow cytometry of human inguinal lymph node cells also showed expression of CXCL13 primarily in PD-1^hi^ CXCR5^hi^ GC-Tfh cells, with few CXCL13^+^ cells among other T cell populations, B cells, or stromal cells [106]. Further, the frequency of GC-Tfh cells in lymph nodes correlates positively with serum CXCL13 levels in both humans and non-human primates, such that the serum CXCL13 level has been suggested to represent a biomarker of total GC activity. If cells produce CXCL13 in order to recruit B cells (which express high levels of CXCR5, the only known receptor for CXCL13), then CXCL13-producing T cells (GC-Tfh cells and others) are prime candidates to interact with B cells in various settings.

While GC-Tfh cells are a major source of CXCL13 in lymph nodes, there are accumulating data that non-Tfh cells with varied surface phenotypes can produce CXCL13 at different sites. In Sjogren's syndrome patients, CCR9^+^ CXCR5^−^ T cells from the blood produced CXCL13 (in addition to IL-21 as described above), albeit at lower levels than was produced by CXCR5^+^ cells [83]. CXCL13 production from CD4^+^ T cells in RA synovium was first described by Manzo et al., who noted CXCL13^+^ CD4^+^ T cells both in areas of large lymphoid aggregates and also in areas with smaller aggregates or diffuse lymphocyte infiltration [107]. Flow cytometry of synovial fluid T cells revealed that most CXCL13^+^ T cells are memory CD4^+^ T cells that lack CXCR5 and contain little Bcl6. These CXCL13^+^ T cells were often CD27 negative, suggesting a chronically activated or terminally differentiated phenotype [107]. An independent study identified the same population of CXCL13^+^ CD4^+^ T cells with little CXCR5 or Bcl6 expression, but with high expression of PD-1 [108]. Detailed assessments by mass cytometry, RNA-seq transcriptomics, and *in vitro* B cell-helper assays then defined this synovial T cell population as Tph cells [50].

Single cell RNA-seq analyses of synovial tissue have recently highlighted Tph cells as the predominant source of CXCL13 in rheumatoid synovium. The vast majority of CXCL13 signal in RA synovium detected by single cell RNA-seq is in the Tph cell population, with little CXCL13 detected in synovial fibroblasts, macrophages, vascular cells, or B cells [109]. This powerful analysis provides a high-resolution view of the potential cellular sources of CXCL13 in the tissue, which can be difficult to discern from immunohistochemical analysis given that CXCL13 can deposit on matrix components once secreted [40]. CXCL13 has emerged as a promising biomarker in RA, with its level in the synovium associated with the presence of synovial lymphoid aggregates, autoantibody positivity, and erosive disease [110, 111]. In this context, it is remarkable that the majority of this CXCL13 appears to come from infiltrating Tph cells [109]. Notably, CXCL13 production by human B cell-helper T cells stands in stark contrast to the murine Tfh cells, which do not make CXCL13. Rather, stromal and parenchymal cells appear to be the major producers of CXCL13 in murine tissues, stimulated by lymphotoxin α/β and TNF in SLOs or IL-17A in inflamed tissues [112, 113].

Single cell RNA-seq analyses appear to be particularly sensitive in identifying CXCL13^+^ T cell populations, perhaps because CXCL13 mRNA transcripts are expressed at very high levels in restricted subpopulations of cells [50, 109]. RNA-seq of single cell T cells sorted from hepatocellular carcinoma samples identified an easily distinguished CXCL13^+^ CD4^+^ T cell population isolated from tumor tissue [114]. The chemokine receptor expression on this T cell population (e.g., CXCR5) was not evaluated in this study; however, an independent study of T cell infiltrates in hepatocellular carcinoma identified a prominent IL-21-producing T cell population that accumulates within the peritumoral stroma [115]. These cells, which are CXCR5^−^ PD-1^low^, comprise ~10% of tumor CD4^+^ T cells, and their frequency correlates positively with plasma cell infiltrates and negatively with overall survival [115]. Whether the CXCL13^+^ cells seen by RNA-seq and IL-21^+^ T cells detected by flow cytometry represent the same T cell population in hepatocellular carcinoma will be of interest to resolve.

Detailed studies of breast cancer tissue have also revealed a population of infiltrating CXCL13^+^ CD4^+^ T cells [105, 116]. In breast cancer tissue, CXCL13^+^ CD4^+^ T cells accumulate either within small B cell aggregates, adjacent to B cell follicles, or occasionally within B cell follicles [105, 116]. These CXCL13^+^ T cells display a PD-1^hi^ ICOS^mid^ CXCR5^−^ surface phenotype and express high Tbet but relatively low Bcl6 levels compared to tonsil GC-Tfh cells [105]. In addition to elevated CXCL13, these cells showed high expression of IL-21, IFN-γ, and IL-10. The presence of CXCL13^+^ T cells in breast cancer tissue was found to be a good prognostic sign, associated with a higher rate of disease-free survival [105]. While most CXCL13^+^ T cells in breast cancer samples are CD4^+^ T cells, a subset of tumor-infiltrating CD8^+^ T cells was also noted to produce CXCL13 and reside near B cell follicles, albeit with lower CXCL13 expression than in CD4^+^ T cells [105]. CXCL13 expression has also been observed in CD103^+^ CD8^+^ T cells in ovarian cancer tumors [117]. It will be interest to evaluate further the relative contributions, and functional overlap, of CD4^+^ and CD8^+^ T cells that produce CXCL13. Notably, CD4+ T cells with B cell-helper features, including high expression of PD-1, ICOS, and CXCL13, are enriched in multiple solid tumors and in murine tumor model, and these cells are preferentially expanded by anti-CTLA4 but not anti-PD-1 therapy [118–120]. Clarifying the beneficial vs. deleterious roles of these cells in anti-tumor immune responses will be important in order to utilize this population as a predictive biomarker.

## Regulation of CXCL13 production in T cells

Compared to other effector functions, relatively little is known about the regulation of CXCL13 production in human CD4^+^ T cells. Triggering of the T cell receptor plus the CD28 costimulatory receptor on synovial CD4^+^ T cells augments CXCL13 secretion [107]. Secretion of CXCL13 by synovial T cells appears relatively long-lived and can be sustained for several days by the addition of TNF + IL-6 to *in vitro* cultures [108]. CXCL13-producing synovial CD4^+^ T cells show a stable phenotype and are able to retain the ability to produce CXCL13 for several weeks in culture [108]. Interestingly, overexpression of Bcl6 enhances production of CXCL13 production by human tonsil CD4^+^ T cells, suggesting that Bcl6 controls not only the localization of Tfh cells, but also their ability to direct co-localization of B cells [13]. Recruitment of B cells through CXCL13 production may act in synergy with upregulation of molecules involved in the T cell-B cell interaction, such as CD40L, PD-1, and ICOS, by Bcl6 [13].

Signals that induce naïve CD4^+^ T cells to acquire the ability to produce CXCL13 are not well defined. TGF-β appears important, as treatment of naïve CD4^+^T cells with TGF-β during *in vitro* stimulation promotes CXCL13 production [105, 121]. Activin A, which signals through the Activin A receptor to activate Smad2-Smad3 pathways in a manner similar to TGF-β, also promotes CXCL13 production [122]. CXCL13 production has also been detected from *in vitro* differentiated Th17 clones, which may be a consequence of the TGF-β used in the Th17 differentiation culture conditions [123]. In addition, as with differentiation of mouse Tfh cells, IL-2-induced activation of STAT5 inhibits the CXCL13-producing phenotype in human CD4^+^ T cells [105, 121].

## TPH cells as potential instigators of ELS

**Figure 2:**
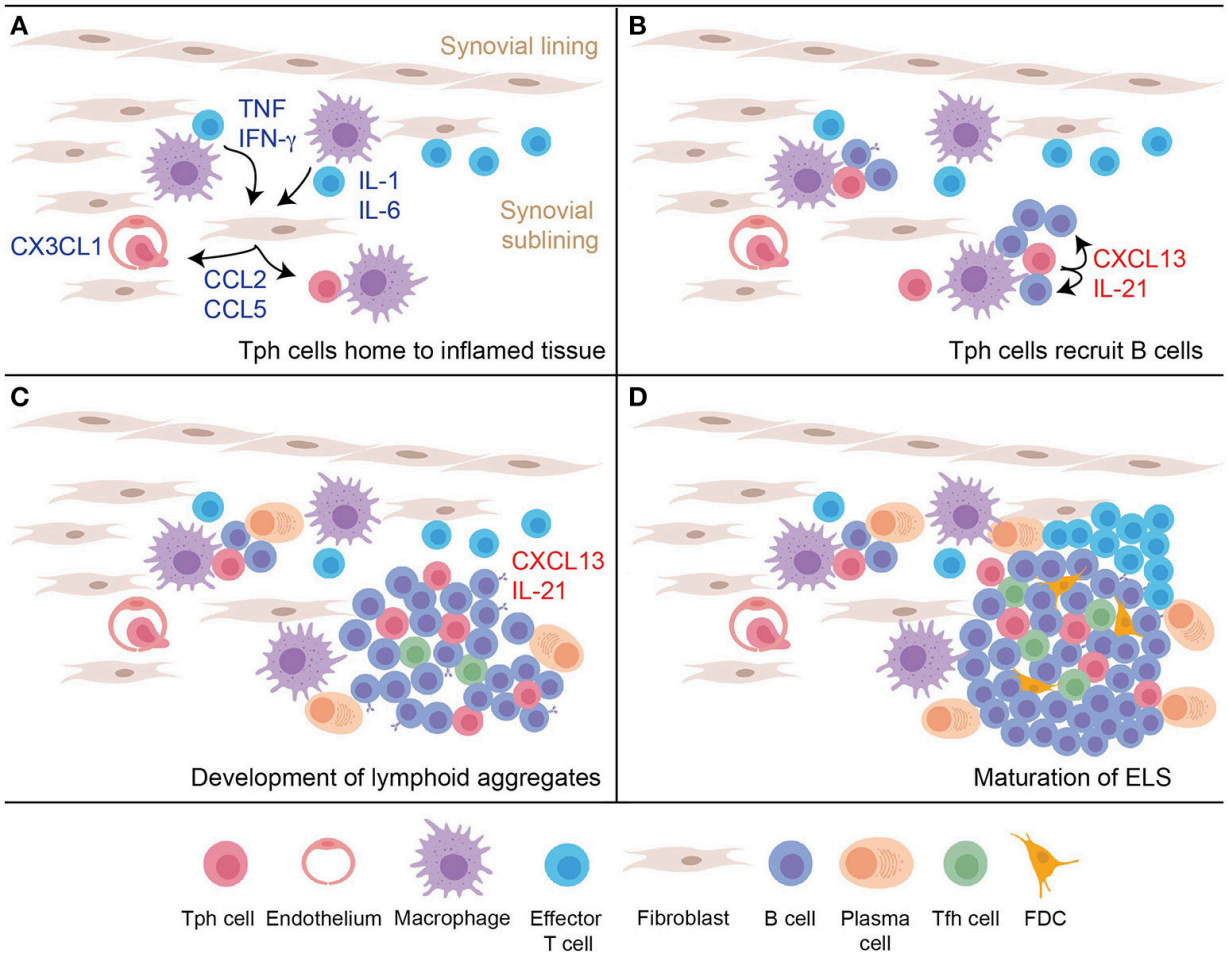
A model for nucleation of ELS in inflamed tissues by Tph cells. **(A)** An inflammatory response in the peripheral tissue induces production of chemokines and additional signals to recruit peripheral-homing Tph cells. **(B)** After infiltrating the tissue, activated Tph cells produce CXCL13 to recruit B cells, and produce IL-21 to promote B cell activation and survival. **(C)** Continued activation of Tph cells and interaction with B cells leads to development of loose lymphoid aggregates, which support some plasma cell differentiation. Tfh cells begin to be recruited into these aggregates in part due to CXCL13 production. **(D)** Ongoing T cell-B cell interactions and CXCL13 production leads to further maturation of the lymphoid aggregate into organized follicles that acquires features of GC, including accumulation of FDC.

Production of CXCL13 by T cells that infiltrate an inflamed peripheral tissue may be a key step in the initiation of ELS formation, providing an early stimulus to recruit CXCR5^+^ B cells (Figure 2). Proposed here is a model in which Tph cells infiltrate a site of peripheral inflammation, drawn in by chemokines such as a CCL2, CCL5, and CX3CL1. Upon encountering antigen plus inflammatory cytokines within the tissue, Tph cells produce large amounts of CXCL13, which help recruit CXCR5^+^ B cells into the tissue and draw them into close proximity with the Tph cells. This process may induce formation of the small lymphocyte aggregates frequently observed in inflamed tissues. Recognition of antigen presented on antigen presenting cells within the tissue, including perhaps on recruited B cells, also induces Tph cell production of IL-21, which helps drive maturation and survival of local B cells. If the reaction occurs vigorously enough, then continued production of CXCL13 and IL-21 will recruit and sustain additional B cells and Tfh cells, as well as eventually FDCs, to yield development of mature ELS. In this model, disrupting the function of Tph cells may abrogate formation of both small and large lymphocyte aggregates within inflamed tissues.

## Questions raised

Many questions remain to understand the range of B cell-helper T cells that drive B cell responses in peripheral tissues. T cells with Tfh-like features have been identified in target tissues in several diseases and models beyond those discussed here, including lupus, autoimmune hepatitis, primary biliary sclerosis, systemic sclerosis, Hashimoto's thyroiditis and several others [37, 124–127]. Detailed phenotypic analyses of these cells in different inflammatory conditions will be of tremendous interest in understanding the features of B cell-helper T cells across diseases. For conditions in which both Tfh cells and Tph cells accumulate in inflamed tissues, the more challenging question will be to determine the relative roles, and potentially distinct functions, of these two B cell-helper populations in the involved tissue.

The developmental relationship between Tfh cells and Tph cells is also a key topic to be clarified. It is possible that a subset of Tfh cells differentiate into Tph cells during the GC response. Alternatively, Tph cells may derive from peripheral effector cells that acquire B cell-helper function. Sorted human CXCR5+ Tfh cells and CCR2+ Tph cells remain relatively distinct after short-term *in vitro* stimulation [50]; however, the developmental relationships between these cells may be most definitively addressed in murine models. In addition, it will be of interest to consider where Tph cells fall within the range of B cell-helper T cells observed in SLOs, which include GC-Tfh, pre-Tfh, and extrafollicular helpers. For example, do these populations comprise a spectrum of B cell-helper cells distinguished by migratory programs, with Tph cells on one far end and GC-Tfh at the other? Additional questions raised by these observations include: Do Tph cells have a role in protective immune responses, or do they primarily mediate chronic, pathologic T cell-B cell interactions? Can Tph cells or peripheral T cell-B cell interactions be specifically targeted to blunt pathologic inflammatory responses?

## Conclusion

While much emphasis has been placed on the central role of Tfh cells in providing B cell help, the phenotypes of B cell-helper T cells in inflamed tissues can differ substantially from Tfh cells. A focus on CXCR5^+^ and Bcl6^hi^ T cell populations may miss large populations of B cell-helper T cells in target tissues, such as the PD-1^hi^ CXCR5^−^ Tph cell population in RA synovium. A broad assessment of T cells that produce the relevant effector molecules and demonstrate B cell-helper function, aided by high-dimensional analyses such as mass cytometry and RNA-seq, will provide a more complete understanding of the T cell-B cell interactions that promote immune-mediated tissue inflammation and the development of ELS in chronically inflamed tissues.

## Author contributions

The author confirms being the sole contributor of this work and approved it for publication.

### Conflict of interest statement

DR is an inventor in a patent submitted on Tph cells.
